# Inequality in Public Health Spending and Access to Healthcare Services in Zimbabwe: A Cross‐Sectional Study

**DOI:** 10.1002/hsr2.71634

**Published:** 2025-12-21

**Authors:** Abigail Chari, Dieter von Fintel, Ronelle Burger

**Affiliations:** ^1^ Research on Socioeconomic Policy, Department of Economics Stellenbosch University Stellenbosch South Africa; ^2^ ODI Global Lilongwe Malawi; ^3^ Institute of Labor Economics (IZA) Bonn Germany

**Keywords:** access to healthcare services, inequality in healthcare services, public health spending, Zimbabwe

## Abstract

**Background and Aims:**

Access to healthcare services is a public health challenge worldwide. Although governments continue to channelscarce resources to enhance service provision, the World Health Organization estimates that about half the world's population lacks essential access. This global context underscores persistent gaps even where investments are made. Public health spending often fails to achieve its aim of helping poor and vulnerable people, creating a critical disconnect between resource allocation and actual service reach. This study examines inequality in public health spending and access to healthcare services in Zimbabwe.

**Methods:**

The study achieves the objective using 2017 government health expenditure from the Ministry of Health and Child Care and data from the 2017 Prices, Income, Consumption, and Expenditure Survey. To ensure consistency between spending patterns and household experiences, the empirical model is based on the concentration index and re‐centered influence functions. The study estimates inequality in public health spending using the standard concentration index, while Erreygers concentration index measures the inequality in access to healthcare services. Oaxaca–Blinder‐RIF decomposition is also used to decompose the differences in the concentration index between urban and rural populations.

**Results:**

The results show that, on average, public health spending in Zimbabwe is pro‐poor for public clinics and pro‐rich for public hospitals. This divergence reflects how different tiers of the health system serve different socioeconomic groups. Despite benefits from government health spending, inequality in the availability and affordability remains a problem, with the affluent continuing to benefit from well‐resourced facilities. Affordability and availability of healthcare services are pro‐rich, much greater in urban than in rural areas. Moreover, the decomposition analysis highlighted that inequality in the affordability and availability between urban and rural populations is largely not explained by the traditional variables, suggesting the influence of deeper systemic and institutional factors.

**Conclusion:**

Zimbabwe needs to aim for universal health coverage, with good quality, affordable care for all, regardless of geographical location or socioeconomic status. Achieving this requires not only sustaining but strategically strengthening current efforts. There is room to improve and augment efforts to achieve universal health coverage without leaving anyone behind. Broader health financing mechanisms and infrastructure investments should be explored to bridge the gap in access to services. Policymakers should, therefore, improve resource allocations in the health sector to achieve equality in government spending and access to healthcare services, ensuring that investment translate into equitable health outcomes for all.

**Abbreviations**
CI: concentration index
PICES: Prices, Income, Consumption, and Expenditure Survey
RIF: Recentered Influence Function
WHO: World Health Organization
ZIMSTAT: Zimbabwe National Statistics Agency

## 1 Introduction

Understanding the causes of inequality in the health sector is crucial to making informed decisions and accelerating universal health coverage. Disparity in access to healthcare services is preventable and unjust, and disadvantages vulnerable and marginalized groups. Although access to healthcare services is deemed everyone's right, the World Health Organization (WHO) estimates that approximately half the world's population does not have access to essential healthcare services; without remedial action, this share is expected to reach two‐thirds by 2030 [1]. This global situation underscores the urgency for Zimbabwe to understand and address its own patterns of inequality. Some reasons for weak access to healthcare services are long distances to health facilities, high service and transport costs, long waiting times, and the opportunity cost of accessing the services. To attain universal health coverage, Zimbabwe's health sector needs to understand the factors causing inequality.

Access to healthcare services is a problem in Zimbabwe [2, 3, 4], especially given that the country has a large disease burden, including a high prevalence of HIV (12.7%) and malaria (29%) and a higher incidence of TB than other sub‐Saharan African countries (210/100,000) [5]. Together, HIV, TB, and malaria are responsible for about one‐fifth of morbidity and mortality in Zimbabwe, and they are significant contributors to inpatient and outpatient treatment [6, 7]. This heavy disease burden magnifies the consequences of unequal access and reinforces the need for targeted interventions.

Amid high burden of disease, the country is overwhelmed by widespread poverty, costly services, and long distances to health facilities that oblige people to make trade‐offs between healthcare and other basic services, and often forgo the medical attention they need [8]. Not consulting health workers when ill may result in high treatment costs in the long run; if treatment is sought at a later stage, the person's health status may have deteriorated significantly, which may result in death [9, 10]. Also, as Gilson and McIntyre [11] and Mangundu et al. [3] note, people tend to substitute conventional healthcare with unconventional alternatives, such as using herbs, previously unused drugs, and drugs borrowed from neighbors. These problems are concentrated amongst the poor, who represent a greater share of health needs proportional to their share of the population. Differences in development between rural and urban areas worsen the disparity in access to healthcare services [12]. The rural areas are underdeveloped and suffer from rudimentary infrastructure and shortages of health workers, drugs, and medical equipment [13]. These geographic and socioeconomic barriers further connect structural inequality to health outcomes, reinforcing unequal access to services. According to the Ministry of Health and Child Care [14], introducing primary healthcare after independence in 1980 improved the availability of healthcare resources in Zimbabwe. This improved healthcare coverage and reduced inequalities in access to healthcare services. Removing user fees for pregnant women's and children's healthcare services at the point of care also improved access to these services [14]. However, access to healthcare services for all remains a public health challenge in Zimbabwe, indicating that earlier gains have not translated into universal equity in access to services.

To improve healthcare service provision, governments have to manage scarce resources effectively to support the health sector. Public health spending aims to benefit the needy and reduce inequality in access to healthcare services, helping the poor and vulnerable to improve their health outcomes, productivity, and living standards. But in developing countries, health systems are almost universally inequitable, with the affluent benefiting more than the poor [15]. This has been noted by Akazili et al. [16] in Ghana, Chuma et al. [17] in Kenya, Ataguba and McIntyre [18] in South Africa, Mtei et al. [19] in Tanzania, O'Donnell et al. [20] in Vietnam, O'Donnell et al. [21] in Asia, Chakraborty et al. [22] in India, and Shamu et al. [23] in Zimbabwe. However, a pro‐poor benefit incidence was found by Rudasingwa et al. [24] in low‐level health facilities in Zambia, and Van der Berg [25] and Burger et al. [24] in both public clinics and public hospitals in South Africa, perhaps because the affluent in South Africa have private medical insurance coverage and tend not to use public facilities. Additionally, Van der Berg [25] and Burger et al. [26] focused on primary healthcare services targeted at poor households. Thus, the inequality found in many studies of developing countries indicates a need to investigate further the conditions under which health services can be pro‐poor.

Several studies have been carried out on public health expenditure and access to healthcare services [27, 28, 29]. Shamu et al. [23] investigated public health financing in Zimbabwe using 2010 national health accounts data, while two other studies on inequality in access to healthcare in Zimbabwe, Chari and Gangaidzo [30], Makate and Makate [31] and Lukwa et al. [32], are specifically about mother and child healthcare access. However, these studies addressed only public health expenditure or access, not both. This separation leaves a critical gap in understanding how spending patterns translate into service access.

Analyzing the relationship between expenditure and access will better inform effective and equal resource allocation to achieve universal health coverage and help inform reforms to reduce health inequalities in low‐ and middle‐income countries. To bridge the gap in the literature, our study examines unequal access to healthcare facilities and public health spending in Zimbabwe. We therefore explored inequality in health access for the general population, a less targeted approach where the inequality is expected to be greater. This approach also aligns with the government's aim of achieving equal access to healthcare services for everyone, not only for specific groups. We used a more recent dataset, the 2017 Prices, Income, Consumption and Expenditure Survey (PICES) and 2017 government health expenditure, to determine the disparities in the health sector. Using the concentration indices (CI), we found that inequalities in public spending and access to services exist in the health sector in Zimbabwe, thus providing the evidence required to understand and address the interconnected inequalities highlighted in this study.

## 2 Methods

### 2.1 Data and Variables

#### 2.1.1 Government Health Spending

The Department of Finance and Administration of Zimbabwe's Ministry of Health and Child Care (MoHCC) collects data on health expenditure, both recurrent and capital, from all 10 of the country's provinces: Bulawayo, Harare, Manicaland, Mashonaland Central, Mashonaland East, Mashonaland West, Masvingo, Matabeleland North, Matabeleland South, and Midlands. These data represent government health expenditures channeled towards public clinics and hospitals. In this study, we used MoHCC data for recurrent expenditure, which benefits only the current generation, rather than capital expenditure, which benefits many generations. For example, medicines bought today are used by the current generation, but hospitals built today are also used by future generations.

MoHCC aggregated the data for government health expenditures for public hospitals and clinics at the district level. The MoHCC uses the ratio 4:6 to allocate health expenditures between public clinics and hospitals in Zimbabwe. We used this ratio to determine the distribution of public health expenditures across these two facility types. MoHCC tends not to apply this ratio during disease outbreaks but allocates funds to contain the epidemic. However, in this study, the disaggregation using the ratio was justified, given the stable disease environment during 2017.

#### 2.1.2 Poverty, Income, Consumption, and Expenditure Survey

The PICES gathers information on socioeconomic and demographic characteristics of households and individuals, poverty, consumption, expenditure, and agricultural production. The survey used a sample of 32,256 households, drawn from all administrative districts using a two‐stage sampling technique. Thus, enumeration areas were selected first from rural and urban areas, and households were then allocated to their respective enumeration areas. The data comprised 2304 enumeration areas, which were selected from the 2012 population census data. Of the 32,256 households selected for the interview, 31,198 were successfully interviewed, yielding a 96.7% response rate. Each district had 36 enumeration areas and 508 households. Harare and Bulawayo, the largest administrative districts and urban provinces, had double the enumeration areas and households to ensure representativeness at the district level.

The 2017 PICES data represent Zimbabwe's population at the district level. We weighted our sample to ensure population representativeness. We selected individuals who had reported any illness in the previous 30 days. This gave us a sample of 14,320 individuals out of the 136,799 in the survey. Participants were asked if they had been ill in the previous month and if they had used healthcare services during that time. The questions were: “Was (name) ill/sick in the last 30 days?” and “Did (name) visit a healthcare provider for services in the last 30 days?” This tended to eliminate those who had been ill at least one month before the survey and those with chronic conditions, who may not have responded “yes” to the first question. This survey question may thus have caused an underestimation of illnesses in our analysis. In addition, the data were prone to nonresponse bias, as individuals who had not used healthcare services did not respond to the question about illness, despite being ill at some point. Problems with healthcare access may thus have been underreported. To ensure unbiased and consistent estimates, we therefore used the imputed values of affordability and availability of healthcare services at the cluster level based on information from individuals who had used the services.

Information on the use of healthcare services in PICES was based on whether the individual had been ill the month before the survey. The data comprise only services used when an individual was acutely ill, not those used for chronic conditions and prevention. We therefore aggregated utilization rates per socioeconomic group and district, given this data limitation. The PICES also asks respondents about only one facility visit if an individual had been ill in the 30 days before the interview, although the individual may have made many visits to one facility or visited many facilities. As strongly argued by Alaba and McIntyre [33], general household surveys are prone to the abovementioned data deficiencies, which means they underreport the need and utilization of healthcare services. In addition, the nature of the PICES data made it impossible to classify facilities into usual levels of care, primary, secondary, tertiary, and quaternary, but in this study we were able to disaggregate them into public clinics and hospitals. However, while the data may underestimate the utilization of healthcare services and needs in Zimbabwe, they provide up‐to‐date information we need to analyze the distribution of public expenditure and inequality in access to healthcare services.

To calculate the benefit incidence, we merged the MoHCC health expenditure data with the individual PICES data (the sample of 14,320 individuals), aligning the district health expenditure for public clinics and hospitals to the individuals from the same districts who used the services in the PICES data.

#### 2.1.3 Definition of Variables

##### 2.1.3.1 Benefit Incidence

As noted by O'Donnell et al. [20], the benefit of government spending is determined chiefly by the utilization of healthcare services and the share of government healthcare spending. We assumed that the unit subsidy was constant across healthcare levels and health services.^1^ This assumption implies that the subsidy is different for hospitals and clinics and that the average subsidy does not vary according to the length of a hospital stay or healthcare quality [21].

We calculated the benefit incidence in four steps. First step, we calculated the unit cost of providing healthcare services by dividing the total current government health spending by the total number of users of healthcare services in a district (see [22]). We used current health spending to calculate benefit incidence, as it benefits the current generation, while capital health spending also benefits future generations, as noted earlier. We did not subtract user fees from the unit cost in this analysis, as the amount was too small (less than 1% of total household expenditure) to affect the unit subsidy.

In the second step, we assigned the unit cost to users of public healthcare services per district and facility type (public clinic or public hospital). Users of healthcare services benefit from the in‐kind transfer of government health expenditure [34]. These individuals had used public healthcare facilities in the month preceding the PICES. The third step was to aggregate the users into socioeconomic groups by ranking them from poorest to richest using quintiles, using per capita household expenditure, deflated using the upper poverty line, and Harare as the area and June 2017 as the reference period [8].^2^

In the fourth and final step, we calculated the benefit incidence of government health spending as follows:

(1) $$X_{\mathrm{jp}}=\sum_{i=1}^{n}\frac{S_{\mathrm{ip}}}{H_{\mathrm{ip}}}\times H_{\mathrm{ijp}}=\sum_{i=1}^{n}\frac{\mathrm{Hijp}}{\mathrm{Hip}}S_{\mathrm{ip}}=\sum_{i}^{n}h_{\mathrm{ijp}}S_{\mathrm{ip}},$$

where *X*_*jp*_ was the total subsidy socioeconomic group *j* in district *p* receives as government health expenditure, *H*_*ijp*_ was the number of health visits by socioeconomic group *j* to a facility at level *i* in district *p* (public clinics and hospitals), *H*_*ip*_ was the total number of visits (for all the groups) to level *i* facility in district *p*, and *S*_*ip*_ was the subsidy, that is the total government expenditure less the household spending on healthcare services in district *p* (out‐of‐pocket payments). $\frac{S_{i}}{H_{i}}$ was the unit subsidy of the government expenditure at level *i* across all the individuals, and *h*_*ijp*_ was the share of utilization of facility‐level *i* by a socioeconomic group *j* in district *p*.

We did not do the target distribution in this study due to a lack of available data.

##### 2.1.3.2 Access to Healthcare Services

Affordability is an access dimension defined as the extent to which individuals can pay for healthcare services [35]. We used affordability as a dependent variable measured at the cluster level. We calculated the average health expenditure within a cluster as the total health expenditure paid divided by the total number of individuals who were ill in that cluster in the month before the survey. This assumed that individuals in the same enumeration area would be more likely to suffer from almost the same acute illnesses and pay almost the same healthcare costs. The clustered health expenditure was then imputed to individuals within different clusters. In the survey, health expenditure was imputed following ZIMSTAT's [8] computation of school fees for students with missing information. We used cluster‐level health expenditure as a proportion of total per capita nonfood consumption expenditure to measure the affordability of healthcare services. A value of less than 40% of cluster‐level health expenditure as a proportion of total per capita nonfood consumption expenditure is considered affordable, and thus not catastrophic [36]. We assigned a value of *1* when services were affordable and *0* otherwise.

We used the other access dimension, availability, as a dependent variable measured using distance to the health facility, as done by Perry and Gesler [37] and Rosero‐Bixby [29]. The PICES data include the distance traveled by individuals to the nearest health facility when ill, meaning that the question is only posed to those who had consulted a health worker in the month before the survey. We therefore used the average cluster distance, as used to measure availability, if individuals in the same cluster tended to use the same facility. Average cluster distance was measured as the sum of the distance reported by the individuals treated in a cluster divided by the total number of households in the area. If individuals had to travel, at most, 5 km to the facility, we considered the service available. We assigned a value of *1* for individuals with available services, and *0* otherwise.

**Figure A1:**
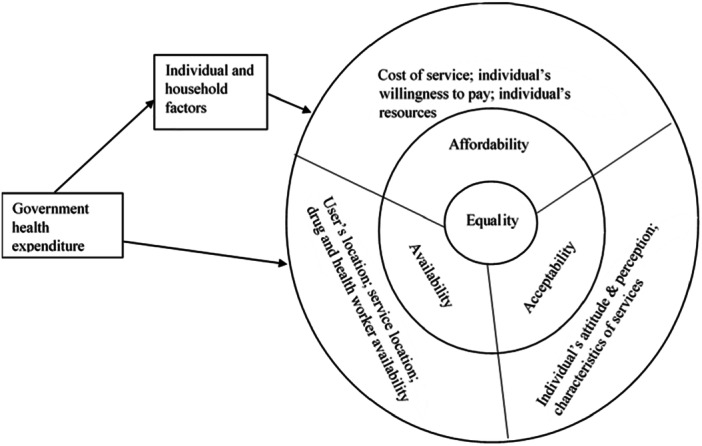
Conceptual framework of access to healthcare services. *Source:* Adapted from Peters et al. [63].

The contributing factors, taken from the conceptual framework in Figure A1, were gender, age, education, marital status, household size, transport availability, health insurance, and urban–rural differences. *Gender* was a binary variable, represented by *0* for *Female* and *1* for *Male*. *Age* was a continuous variable measured in years. These variables were included because men and younger individuals are more likely to have better access to healthcare services than women and older individuals. *Education* was a categorical variable, coded *0* for *No* and *Primary education*, *1* for *Secondary education*, and *2* for *Tertiary education*. *Marital status* was also a categorical variable, represented by *0* for *Never married*, *1* for *Married or cohabitating*, and *3* for *Divorced or widowed*. *Household size* was the number of individuals in a household. It was assumed that being educated improves access to healthcare services [27]. *Transport availability* was represented by owning or having free access to a motor vehicle or motorcycle, which improves access to healthcare services [28]. *Health insurance* was a dummy variable, *1* for *Yes* and 0 for *No*. It was assumed that having health insurance coverage improves access to healthcare services. Given the *difference between urban and rural development*, urban populations are more likely than their rural counterparts to access healthcare services when needed [27, 28]. *Urban–rural differences* were a dummy variable, 1 for rural and 0 for urban. Subgroup analyses by urban–rural differences were exploratory and should be interpreted with caution.

### 2.2 Estimation Techniques

#### 2.2.1 Concentration Indices

Several methods can be used to measure inequality, but it is crucial to choose an approach that shows the socioeconomic dimensions of health, represents the experiences of the entire population, and is sensitive to changes in the distribution of resources [38]. The CI satisfies these requirements and has been widely used, for example, in the analyses of Akazili et al. [16], Burger et al. [26], and Shamu et al. [23]. We therefore used the CI, disaggregated by urban and rural areas, to examine the origins of inequality in the Zimbabwean health sector.

The CI reflects the gap between an equal allocation (a 45° line, the equality line) and the concentration curve. The CI lies between −1 and +1. A positive value of the CI was interpreted as pro‐rich distribution of resources, with the concentration curve lying below the equality line. A negative value of the CI was interpreted as pro‐poor distribution, with the concentration curve lying above the equality line. The larger the absolute value of the CI, the greater the inequalities in benefit incidence and access.

The standard CI was specified as follows:

(2) CI=2/μcov(h,r),

where CI was the standardized CI, *h* was the healthcare variable (*Benefit incidence*), *μ* was the *Healthcare* variable's mean, and *r* was the *i*th‐ranked individual in the socioeconomic distribution from the poorest to the richest.

Because the *Availability* and *Affordability* predictors are binary, we used the Erreygers [39] CI to measure inequality in access to healthcare services. The Erreygers CI is mainly used for binary variables and is an improved version of standard CI. Furthermore, the CI is only appropriate when the *Healthcare* variable used has a lower and upper bound [39, 40]. Given the binary nature of the *Healthcare* variable, the lower bound was *0*, and the upper bound was *1*.

The Erreygers CI addresses the shortcomings of the standard CI by showing the concentration of healthcare in a specific socioeconomic group [41].

The Erreygers CI was computed as follows:

(3) $$E_{C}=\left(4\mu_{y}/b-a\right)\mathrm{CI},$$

where CI was the standardized concentration index, and *a* and *b* were the upper and lower bounds, respectively, of the *Healthcare* variable. The size and extent of *E*_*C*_ showed the strength and variability of access to healthcare services. The Erreygers CI incorporates the “mirror property” assumption, which states that health inequalities should mirror ill‐health variations that differ from the standard CI [42, 43]. The Erreygers CI is not overdependent on the mean of the healthcare variable, as in the case of the standard CI.

#### 2.2.2 Re‐Centered Influence Functions and Oaxaca–Blinder‐RIF Decomposition

We used re‐centered influence functions (RIFs) to explain the contribution of explanatory variables to inequality in access to healthcare services. The main aim of decomposition analysis is to evaluate the distribution of the outcome variable as explained by changes in the explanatory variables [20]. We used the RIF decompositions to examine how urban–rural differences contributed to inequality in access to healthcare services. This contribution is a major factor, given the very different levels of development between rural and urban areas in Zimbabwe [2]. The decomposition of inequality is crucial for policymakers to understand the sources of inequality. This analysis was done for the inequality in access to healthcare services, not inequality in public spending, due to data unavailability on factors affecting inequality in public spending.

RIFs are deemed more appropriate for bivariate rank‐dependent indices, given that they decompose all forms of CIs [44, 45]. The other reasons why RIFs are more relevant for decomposition analysis are that they are flexible, easy to interpret, and clearly show the contribution of the covariates to inequality, which is essential for decision‐making [44, 45]. Rios‐Avila [45] and Firpo et al. [46] state that the simple approach to estimating RIF regressions is to assume linearity in parameters and the error term and linearity in the RIF's dependent variable and independent variables. Thus, the change in the independent variables can explain a slight change in the CI [44]. Rios‐Avila [45] notes that the direction of change can be interpreted regardless of the magnitude when using RIFs. We therefore used RIFs to decompose contributing factors to inequality. This examines the associations, not the causal relationships. The RIF was specified as follows:

(4) $$\mathrm{RIF}\left\{y,v\left(F_{Y}\right)\right\}=X^{‘}\beta +\varepsilon_{i},E\left(\varepsilon_{i}\right)=0,$$

where $\mathrm{RIF}\left\{y,\upsilon \left(F_{y}\right)\right\}$ was the dependent variable used for each observation $y_{i}$ for RIFs, y was inequality in the availability and affordability of healthcare services, X was the matrix for independent variables, β was the parameters to be estimated, and $\varepsilon_{i}$ was the error term.

We also used the Oaxaca–Blinder‐RIF decomposition, which is the combination of RIF regression and Oaxaca–Blinder decomposition [47], to decompose the differences in CIs between urban and rural populations into explained and unexplained components (see [48]). The unexplained component (structure effect) shows the differences in inequality due to differences in the coefficients of the characteristics between urban and rural populations, while the explained component (composite effect) represents differences in inequality between urban and rural populations due to differences in characteristics [45, 46]. According to Rios‐Avila [45] and Nghiem et al. [48], the Oaxaca–Blinder‐RIF decomposition is simple to implement, provides a comprehensive contribution of each independent variable to differences, and can be applied to other RIF functions. Inequality in affordability and availability was decomposed as follows:

(5) $$v(F_{Y}^{u})-v(F_{Y}^{r})=\underset{\mathrm{Explained}}{\underbrace{{\left({\bar{X}}_{u}-{\bar{X}}_{r}\right)}^{\prime }{\hat{\beta }}_{p}}}+\underset{\mathrm{Unexplained}}{\underbrace{{\bar{X}}_{u}\left({\hat{\beta }}_{u}-{\hat{\beta }}_{p}\right)+{\bar{X}}_{r}^{\prime }\left({\hat{\beta }}_{p}-{\hat{\beta }}_{r}\right)}},\;$$

where ${v(F}_{Y}^{u})$ and ${v(F}_{Y}^{r})$ were availability and affordability CIs indices for urban and rural populations, respectively, and ${\hat{\beta }}_{u}$, ${\hat{\beta }}_{r}$, and ${\hat{\beta }}_{p}$ were the parameters to be estimated for urban, rural and pooled populations, respectively. The analysis was conducted using STATA 17 software.

## 3 Results

Table 1 summarizes the means of rural and urban populations for relevant demographic and socioeconomic variables. *T* statistics were used to determine whether there were significant differences between the means for the rural and urban subpopulations.

**Table 1:** Descriptive statistics.

	Urban		Rural			
Variables	Mean	SD	Mean	SD	Difference	*t* statistic
Gender (ref: female)	0.388	0.487	0.407	0.491	−0.019	(−1.570)
Age	27.462	20.636	30.808	22.517	−3.345***	(−6.345)
Marital status						
Never married	0.284	0.451	0.245	0.430	0.039***	(2.003)
Married or cohabitating	0.513	0.500	0.526	0.499	−0.013	(−0.609)
Divorced or widowed	0.202	0.402	0.229	0.421	−0.027	(−0.019)
Household size	4.542	1.930	5.223	2.215	−0.682***	(−13.731)
Education						
Primary	0.381	0.486	0.660	0.474	−0.279***	(−13.786)
Secondary	0.533	0.499	0.325	0.468	0.208***	(10.167)
Tertiary	0.086	0.280	0.015	0.122	0.071***	(4.784)
Socioeconomic status						
1	0.015	0.121	0.268	0.443	−0.253***	(−24.496)
2	0.053	0.225	0.252	0.434	−0.199***	(−17.505)
3	0.140	0.347	0.215	0.432	−0.075***	(5.087)
4	0.285	0.451	0.168	0.411	0.117***	(6.829)
5	0.507	0.500	0.096	0.374	0.411***	(22.883)
Per capita nonfood consumption exp	78.619	75.303	25.011	29.000	53.608***	(29.388)
Affordability	0.546	0.498	0.642	0.479	−0.096***	(−7.214)
Availability	0.577	0.494	0.281	0.449	0.296***	(23.490)
Observations	1799		12,521		14,320	

Abbreviation: SD, standard deviation.

*** *p* < 0.01.

The rural areas had a larger share of men, significantly larger household sizes, and a less educated and, as expected, poorer population. In contrast, urban areas had a considerably older population and a higher average monthly nonfood consumption expenditure. The urban areas had a higher availability of healthcare services, but, surprisingly, fewer urban respondents, on average, deemed the health services affordable. Urban populations normally have higher levels of education and greater access to transport, associated with improved access to services. These substantial urban–rural disparities are not only indicators of broader socioeconomic inequality but also potential contributors to unequal access to healthcare services.

The chi‐square of association in Table B1 in the Appendix section shows a significant association between the access to healthcare barriers (affordability and availability) and urban–rural differences.

Table 2 shows the share of respondents who were ill in the 30 days before the survey and the share of those who had consulted a health worker. Overall, 11.1% of the sample reported being ill a month before data collection: 9.5% in urban areas and 11.8% in rural areas. The poor reported less ill health than the affluent, as indicated by 7.1% for the bottom quintile of the urban areas and 9.4% for the top, and 10.5% for the bottom quintile of the rural areas and 15.1% for the top. As can be seen, rural inhabitants reported more illness than their urban counterparts. Of the total sample, 63.7% consulted a health worker at a healthcare facility when ill, with healthcare facilities including public clinics, public hospitals, private clinics, private hospitals, and pharmacies. Thus, almost two‐fifths of Zimbabweans did not consult health workers when ill. Rural inhabitants were significantly more likely than urban inhabitants to seek treatment when ill. In the urban areas, the poorest (46.3%) were less likely than the richest (64.5%) to seek treatment when ill, but in the rural areas, surprisingly, there was no noticeable difference. There was no significant difference between individuals consulting health workers in urban and rural areas by socioeconomic group, except in the fourth quintile.

**Table 2:** Share of people treated when ill.

	Share ill/injured in last 30 days				Share consulted when ill			
Socioeconomic status	Total	Urban	Rural	Urban–rural	Total	Urban	Rural	Urban–rural
1	0.104	0.071	0.105	−0.034***	0.645	0.463	0.648	−0.185
	(0.002)	(0.019)	(0.002)		(0.010)	(0.134)	(0.010)	
2	0.111	0.096	0.112	−0.016***	0.637	0.619	0.639	−0.020
	(0.002)	(0.013)	(0.002)		(0.010)	(0.068)	(0.010)	
3	0.117	0.092	0.124	−0.032***	0.655	0.673	0.673	0.000
	(0.003)	(0.008)	(0.002)		(0.011)	(0.039)	(0.010)	
4	0.119	0.100	0.136	−0.036***	0.602	0.551	0.636	−0.085***
	(0.003)	(0.005)	(0.003)		(0.014)	(0.027)	(0.012)	
5	0.107	0.094	0.151	−0.057***	0.649	0.645	0.648	−0.003
	(0.003)	(0.004)	(0.005)		(0.015)	(0.021)	(0.017)	
Total	0.111	0.095	0.118	−0.023***	0.637	0.614	0.645	−0.031***
	(0.001)	(0.003)	(0.001)		(0.005)	(0.010)	(0.004)	

*Note:* Standard errors are in parentheses.

*** *p* < 0.01.

**Figure 1:**
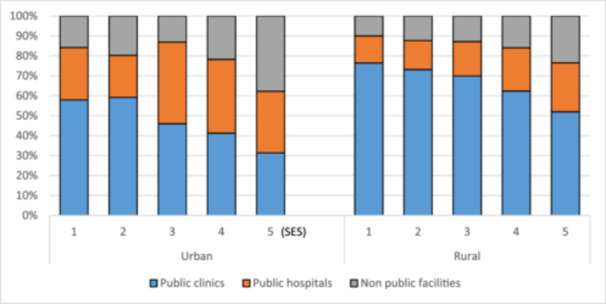
Public health facility utilization by location and socioeconomic status. *Note:* Share of utilization of public clinics and hospitals in urban and rural areas by socioeconomic status.

Figure 1 shows the utilization of public clinics and hospitals by socioeconomic status for urban and rural populations. A socioeconomic gradient is evident in the share using public facilities. The share of the poor using public clinics in rural and urban areas was higher than the share of the affluent using them. The share of the affluent using better‐resourced public hospitals was higher than that of the poor in urban and rural areas. Significantly, more of the affluent use nonpublic facilities than their poor counterparts in urban and rural areas. In addition, more of the population uses public clinics than public hospitals and nonpublic facilities in urban and rural areas.

Table 3 shows the standard CIs for benefit incidence, for public clinics and public hospitals, disaggregated by urban and rural areas. A negative CI indicates that the benefit incidence was pro‐poor. The CI is significantly negative for public clinics (−0.207), but positive for public hospitals (0.298). This suggests that, on average, government health expenditures were pro‐poor for public clinics and pro‐rich for public hospitals.^3^ The CI is significantly negative for public clinics (−0.299) in urban areas but not for public hospitals. The CI is also significantly negative for public clinics in rural areas but not for public hospitals. Thus, the public clinic CI is more negative for urban areas than for rural areas.

**Table 3:** Standard concentration index for benefit incidence.

Facilities	Public clinics	*N*	Public hospitals	*N*
Urban CI	−0.299*** (0.078)	510	−0.101 (0.100)	447
Rural CI	−0.114*** (0.010)	59,641	−0.108 (0.197)	1441
Total CI	−0.207*** (0.012)	6451	0.298*** (0.110)	1888

*Note:* Robust standard errors are in parentheses.

*** *p* < 0.01.

Table 4 shows the Erreygers CIs for access to healthcare services for rural and urban areas regarding affordability and availability of services. Positive CIs show that access to healthcare is pro‐rich, negative that it is pro‐poor. Affordability is pro‐rich, favoring the urban (0.234) rather than rural (0.165) areas, as the former benefit from more affordable services. Availability is pro‐poor, with large differences between the urban and rural areas, resulting in a positive and significant overall CI for availability.

**Table 4:** Erreygers concentration index for access to healthcare services.

Predictors	Affordability proportion of health and per capita nonconsumption expenditure < 40%	*N*	Availability distance < 5 km	*N*
Urban CI	0.234*** (0.060)	1593	−0.103** (0.049)	1658
Rural CI	0.165*** (0.024)	11,198	−0.023 (0.024)	11,601
Total CI	0.114*** (0.029)	12,791	0.085*** (0.029)	13,259

*Note:* Robust standard errors are in parentheses.

*** *p* < 0.01.

** *p* < 0.05.

We examined the sensitivity and robustness of these CIs to differences in household sizes and composition, using equivalence scales (OECD‐modified, OECD, and square root scale), which are used for countries without established equivalence scales, such as Zimbabwe [49]. The results in Table 4 using per capita household consumption expenditure were not significantly different from those shown in Tables B2 and B3 in the Appendix, which use equivalence scales.

Table 5 shows how the RIFs varied according to age, gender, rural or urban location, education level, health insurance status, access to transport, household size, and marital status. In the full sample, an increase in the share of the population living in rural areas made affordability more pro‐rich, more people with access to transport and tertiary education made affordability significantly pro‐rich, and an increase in household size made affordability significantly more pro‐poor. Age, gender, secondary education, health insurance status, and marital status were insignificant in terms of affordability. For urban areas, more people with access to transport and health insurance made affordability more pro‐rich, while for the divorced or widowed, affordability was pro‐poor. For rural areas, older individuals and greater household sizes had significant pro‐poor affordability, while men, those with tertiary education, the divorced or widowed, and access to transport had pro‐rich affordability.

**Table 5:** RIF of covariates on inequality in affordability and availability.

	Affordability			Availability		
	(1)	(2)	(3)	(4)	(5)	(6)
Variables	Full sample	Urban	Rural	Full sample	Urban	Rural
Age	0.000	0.001	−0.002*	−0.002**	−0.005**	−0.001
	[−0.005,0.005]	[−0.008,0.010]	[−0.005,0.000]	[−0.006,−0.001]	[−0.010,−0.000]	[−0.002,0.000]
	(0.001)	(0.005)	(0.001)	(0.001)	(0.002)	(0.001)
Gender (male = 1; female = 0)	0.050	−0.015	0.074**	−0.049	−0.167*	−0.010
	[−0.076,0.145]	[−0.234,0.204]	[0.007,0.141]	[−0.161,0.037]	[−0.348,0.014]	[−0.062,0.042]
	(0.038)	(0.112)	(0.034)	(0.030)	(0.092)	(0.027)
Location (rural = 1; urban = 0)	0.135**			0.088*		
	[−0.225,−0.002]			[−0.020,0.180]		
	(0.057)			(0.049)		
Education (ref: primary)						
Secondary	−0.012	−0.140	−0.004	0.002	−0.000	0.003
	[−0.166,0.046]	[−0.365,0.085]	[−0.71,0.063]	[−0.099,0.094]	−0.189,0.189]	[−0.050,0.056]
	(0.037)	(0.115)	(0.034)	(0.031)	(0.097)	(0.027)
Tertiary	0.341***	−0.036	0.447***	−0.056	−0.166	0.215
	[−0.186,0.329]	[−0.365,0.294]	[0.225,0.668]	[−0.225,0.231]	[−0.473,0.140]	[−0.104,0.534]
	(0.127)	(0.168)	(0.113)	(0.113)	(0.156)	(0.163)
Health insurance (yes = 1; no = 0)	−0.040	0.557***	−0.213	0.688***	0.742***	0.643***
	[−0.61,0.063]	[0.212,0.902]	[−0.642,0.215]	[0.272,1.006]	[0.326,1.158]	[0.195,1.091]
	(0.202)	(0.176)	(0.219)	(0.156)	(0.212)	(0.229)
Transport (yes = 1; no = 0)	1.047***	1.255***	0.861***	0.678	1.590***	0.053
	[0.816,1.341]	[0.924,1.586]	[0.438,1.284]	[0.472,1.864]	[1.172,2.007]	[−0.499,0.605]
	(0.220)	(0.169)	(0.216)	(0.428)	(0.213)	(0.282)
Household size	−0.032***	−0.021	−0.016**			
	[−0.044,0.001]	[−0.067,0.025]	[−0.031,−0.001]			
	(0.009)	(0.024)	(0.008)			
Marital status (ref: never married)						
Married or cohabitating	0.009	−0.038	0.044			
	[−0.065,0.174]	[−0.370,0.294]	[−0.059,0.146]			
	(0.057)	(0.169)	(0.052)			
Divorced or widowed	−0.004	−0.315	0.126*			
	[−0.053,0.040]	[−0.783,0.153]	[−0.013,0.266]			
	(0.082)	(0.238)	(0.071)			
Constant	0.141*	0.518***	0.253***	−0.022	0.146	0.018
	[0.050,0.332]	[0.174,0.863]	[0.137,0.369]	[−0.089,0193]	[−0.064,0.355]	[−0.042,0.078]
	(0.081)	(0.176)	(0.059)	(0.056)	(0.107)	(0.031)
Observations	8150	987	7163	10,470	1272	9198
*R* ^2^	0.012	0.023	0.007	0.008	0.033	0.003

*Note:* Robust standard errors are in parentheses.

*** *p* < 0.01;

** *p* < 0.05;

* *p* < 0.1.

An increase in rural population shares made availability significantly more pro‐rich. More people with health insurance coverage made the availability more pro‐rich. An increase in the number of older people made availability more pro‐poor. Education level and transport had no significant influence on availability. For urban areas, more people with health insurance and better access to transport made availability pro‐rich, while an increase in men or older individuals made availability more pro‐poor. For rural areas, gender, access to transport, and educational attainment were insignificant.

Table 6 shows the differences in inequality in affordability and availability of healthcare services in rural and urban areas. Inequality in affordability (0.222) was much greater in urban than in rural areas. This can be attributed to differences in health prices in urban areas, where the richer people mostly live [50]. Inequality in availability (0.047) was much smaller in urban than in rural areas.

**Table 6:** Oaxaca–Blinder‐RIF decomposition of CI in access to healthcare services.

	(1)	(2)
	Affordability	Availability
Urban	0.371[Link], ***	−0.058*
	[0.305,0.438]	[−0.120,0.005]
	(0.034)	(0.032)
Rural	0.149[Link], ***	−0.011
	[0.124,0.174]	[0.032,0.010]
	(0.013)	(0.011)
Total difference	0.222[Link], ***	−0.047
	[0.151,0.293]	[−0.113,0.020]
	(0.036)	(0.034)
Total explained	0.018*	0.015*
	−0.035,0.037]	[−0.016,0.043]
	(0.011)	(0.008)
Total unexplained	0.204[Link], ***	−0.062*
	[0.150,0.292]	[−0.126,0.006]
	(0.038)	(0.035)

*Note:* Robust standard errors are in parentheses.

*** *p* < 0.01

* *p* < 0.1.

The composition effect of inequality in affordability was positive and insignificant. Despite the significant differences between urban and rural areas in inequality in affordability, adjusting rural endowments decreased the difference by only 1.8%. Of the difference in inequality in affordability between urban and rural areas, 20.4% remained unexplained, and this unexplained difference was significantly positive. The composition effect contributed very little to the difference in inequality in availability between urban and rural areas. Availability inequality between the urban and rural populations decreased by 1.5% if the rural endowments were adjusted. The structure effect was significant, showing that 6.2% of the inequality in the availability of healthcare services between urban and rural areas remained unexplained.

## 4 Discussion

Given the need to attain universal health coverage, it is crucial to understand the health sector's inequalities and ensure that the poor and vulnerable benefit from healthcare services. Our analysis identified some contributors to the inequality in access to healthcare services between urban and rural Zimbabwe, which have large social and economic differences.

We found no difference between richer and poorer individuals who had reported illness in the PICES; thus, there was no socioeconomic gradient. This finding was unexpected. Given their worse living conditions and greater disease risk, we expected the poor to be more likely to report illness than the rich [51, 52]. Rossouw et al. [53] found that poor people, having less access to healthcare services, were less likely than the rich to report chronic illness. Ataguba and McIntyre [54] say the share of people who are ill is expected to follow a socioeconomic gradient due to different perceptions of illness.

Almost two‐fifths of Zimbabweans do not consult health workers when ill [8]. This is consistent with earlier findings by Filmer et al. [55], who found a large proportion of people failing to access essential services. We noted that men, the unemployed, and urban populations were less likely to use healthcare services when ill. Reasons for not consulting health workers when ill are expensive services, treatment not being necessary, home treatment, the facility being too far away, a lack of medication, and religious reasons [8]. Poorer people may also be inclined to put off treatment until they are in the later stages of illness [9, 10], or to resort to alternative sources of healthcare, as Gilson and McIntyre [11] and Mangundu et al. [3] noted.

Analyzing benefit incidence, we disaggregated the health facilities by type, that is, public clinics and public hospitals. The estimated CI showed that the poor received more government health spending through public clinics than the rich. This finding is consistent with those of Van der Berg [25] and Burger et al. [26] for South Africa, Rudasingwa et al. [24] for Zambia, and Castro‐Leal et al. [34] for South Africa and Kenya, and also with that of Shamu et al. [23] for Zimbabwe, using the assumption that unit subsidy is constant. We found that the urban population received more from the subsidy than the rural population. This could be because public clinics in urban areas serve a smaller catchment area than those in rural areas. Filmer et al. [55] posited that government spending on primary healthcare is more effective in improving health outcomes than its spending on secondary and tertiary care.

Our results also indicated a pro‐rich inequality in public hospitals. This finding is consistent with those of Castro‐Leal et al. [34], Macha et al. [56], Shamu et al. [23], and Rudasingwa et al. [24], but contrary to those of Van der Berg [25] and Burger et al. [26]. Despite the Zimbabwe government's efforts to improve healthcare access, its health spending continues to benefit the richer people who mostly use public hospitals located mainly in urban areas and receive more of the subsidy [23, 57]. This urban concentration constrains rural access to tertiary care, even as hospital spending takes a large portion of health budgets.

Despite the government spending, Zimbabweans struggle to access healthcare because it is unaffordable, and the facilities are hard to reach. We found that unavailability and unaffordability are major factors causing unequal access to healthcare in Zimbabwe. Government spending aims to be pro‐poor, but it continues to accrue disproportionately to the richer citizens.

The positive CI for affordability in our analysis indicates a pro‐rich distribution of affordability in Zimbabwe, with greater inequality highlighted in urban than rural areas. This supports findings by Burger and Christian [27], Gilson and McIntyre [11], Harris et al. [28], and Zeng et al. [36], and studies on child‐ and maternal health that found these services benefit the rich more than the poor [30, 31, 58, 59]. We found a noticeable difference in the affordability CI for both rural and urban areas, showing that it was pro‐rich in both areas, but that the inequality was more pronounced in the urban areas.

The RIF decomposition of affordability indicated that tertiary education, health insurance, and access to transport contributed to pro‐rich inequality. Specifically, access to transport increased inequality in affordability, since people with improved access to transport can use services from expensive providers or urban facilities, thus increasing their healthcare options. Conversely, poor individuals cannot afford care even if the transport is available due to indirect costs like income and unaffordable user fees. Furthermore, health insurance exacerbates inequality in the affordability of services, as it often provides limited coverage for the informal sector and poor households, who are less likely to benefit from it.

In rural areas, affordability inequality was also pro‐rich, though less pronounced. The rural poor tend to rely more on public services, which may be cheaper but still unaffordable due to economic constraints. Additionally, rural areas have fewer private providers and often lack alternatives to public services [50]. Moreover, macroeconomic challenges reduce disposable income and force households to choose between healthcare and essential needs like food and shelter [60, 61].

We also found that the availability of healthcare services was pro‐rich in Zimbabwe. The positive CI indicates that affluent people have better physical access to facilities. Availability was significantly pro‐poor in urban areas, showing that the affluent people in urban areas have weak access, which can be attributed to residence in low‐density suburbs located far from public facilities and mainly rely on private services, which are few [8]. Filmer found that richer people tend to substitute private for public facilities based on distance to the public facilities [55]. In rural areas, availability is constrained by poor infrastructure, long travel distances, and sparsely distributed facilities. This aligns with findings from South Africa [28] and other developing countries [2, 3, 52].

Using Oaxaca–Blinder and RIF decomposition, we found that socioeconomic variables such as age, gender, access to transport, health insurance, and tertiary education partially explain the observed inequalities. A large share of inequality remains unexplained, especially due to unavailability. Addressing these factors that apparently bear some responsibility for the differences between rich and poor individuals and regions might go some way towards reducing overall health inequality in Zimbabwe. In this regard, more research on supply‐side and demand‐side factors of inequality is required to determine the factors that should be addressed, as these are outside the traditional set of variables included in household surveys. For example, quality of care, health worker attitude, drug stockouts, and cultural barriers may shape access to healthcare services. Moreover, we only decomposed the inequality in access to healthcare services, and future studies should decompose the inequality in public spending.

Our findings suggest that policy interventions should move beyond targeting affordability through user fee exemptions alone. While exemptions for pregnant women, under‐fives, and the elderly are important, they are insufficient without complementary reforms in service delivery, financing, and outreach. Broader health financing mechanisms and infrastructure investments should be explored to bridge the gap in access to services.

Although the study is important in policy formulation, several limitations are worth noting. The self‐reported survey used is prone to measurement errors from aggregating variables, nonresponse bias, recall bias, and sample selection bias. Considering that the data on some variables was collected for 30 days before the survey, there might be an underestimation of the illness as well as utilization. However, PICES is the only data that provides comparable general service utilization data across the broader population in Zimbabwe.

## 5 Conclusion

Our study provides novel empirical evidence on the nature and drivers of inequality in healthcare access in Zimbabwe. By using benefit incidence analysis with RIF and Oaxaca–Blinder decomposition, the study provides a methodological contribution to equity literature by disaggregating both explained and unexplained components of inequality in access to healthcare services. We found that pockets of inequity in expenditure and access to healthcare services are evident in Zimbabwe, with the urban and affluent benefiting more than the rural and poor populations from better‐resourced facilities. Key contributors to inequality are access to transport, education, and health insurance, yet a large share of inequality remains unexplained, highlighting the role of structural and behavioral factors. This underscores the need for future research to further interrogate factors contributing to inequity in access to healthcare services between urban and rural populations and to add the acceptability dimension of access, which was beyond the scope of this study.

The study offers several policy‐relevant insights, showing that there is room to improve and augment efforts to achieve equity in government spending and access to healthcare services. The Zimbabwe government should focus on the poor and the rural populations, who, despite their acute needs, often struggle to access healthcare services. To improve health equity, the government should adopt a more targeted and redistributive health financing strategy, using poverty and geographic data to guide allocations. There is also a need to improve the physical availability of healthcare facilities, pro‐poor resource allocation frameworks, strengthen user‐fee exemptions, and improve equity monitoring using disaggregated data to attain universal health coverage and Sustainable Development Goals. Such initiatives could improve access to healthcare services for all, ultimately improving health and well‐being.

## Author Contributions

**Abigail Chari:** conceptualization, data curation, formal analysis, methodology, visualization, writing – original draft. **Dieter von Fintel:** conceptualization, formal analysis, supervision, visualization, writing – review and editing. **Ronelle Burger:** conceptualization, formal analysis, supervision, visualization, writing – review and editing.

## Funding

The authors received no specific funding for this work.

## Ethics Statement

Ethical approval for this study was granted by the Stellenbosch University Research Ethics Committee: Social, Behavioral and Education Research (SBER) under project number 24157 per Stellenbosch University's Research Ethics Policy (2013). Data collection procedures for the PICES were approved by the Zimbabwe National Statistical Agency, and written informed consent was obtained from respondents at the beginning of individual interviews in accordance with national ethical guidelines for statistical surveys, ensuring confidentiality, voluntary participation, and informed consent.

## Consent

The authors have nothing to report.

## Conflicts of Interest

The authors declare no conflicts of interest.

## Transparency Statement

The lead author, Abigail Chari, affirms that this manuscript is an honest, accurate, and transparent account of the study being reported; that no important aspects of the study have been omitted; and that any discrepancies from the study as planned (and, if relevant, registered) have been explained.

## Data Availability

The data used for analysis is publicly available on the Zimbabwe National Statistics Agency's website.

## Endnotes

1 Constant unit subsidy refers to the same subsidy amount given for every healthcare service consumed, regardless of the demographic, social, and economic characteristics.

2 We used quintiles to reduce bias, as poor households tend to have more people in the household than rich households, which might result in underestimation of the poor people in the community [20]. The household expenditure comprises food and nonfood expenditure, with the exclusion of intermediate expenditure from consumption expenditure. Furthermore, food expenditure includes all the consumption of the household for 1 month from own production, gifts, purchases, or barter trade.

3 The results show differences between subpopulation CI (positive) and population CI (negative) for benefit incidence in public hospitals. This is referred to as Simpson's paradox, where an association between variables disappears or reverses after separating subpopulations. The difference in the sign of the overall CI and those stratified by urban and rural areas for public hospitals might be attributable to the overlap in the income ranking variable between urban and rural areas [62]. Urban and rural areas contain both affluent and poor people; however, more affluent people reside in urban areas than in rural areas. Since income‐related inequality can be positive or negative, subgroup decompositions also revealed whether the within and between components were of the same type as the overall inequality; that is, whether the within‐location and between‐location indices were of the same sign as the overall index. The within‐location index shows inequality amongst the people in the same subpopulation group (urban or rural), while the between‐location index shows the inequality between two subpopulation samples. This is important, given that the total CI is determined by the within‐location index, between‐location index, and residuals [62]. The residuals, therefore, are nonzero if there is income overlap between subgroups, which contributes to different signs of population and subpopulation CI [62]. This explanation also applies to the results in Table 4.

## Appendix A1 Conceptual Framework

Figure A1 shows a conceptualized framework of access to healthcare services, adapted from Peters et al. [63], with equality at the center. The ultimate goal of the framework is to ensure that everyone has equal access to healthcare services based on their needs and without hindrances. To achieve substantive equality, access to healthcare services is assessed using McIntyre et al.'s [35] three criteria: availability, affordability, and acceptability to users. Access to healthcare services does not have a universally agreed‐upon definition. Penchansky and Thomas [64] define it as the interaction between healthcare providers and users in the health system. We use this conception of access to determine the opportunities available to individuals to use healthcare services when needed.

Access dimensions are, in turn, influenced by individual and household factors and government health spending. Government health spending should aim to ensure that everyone can access the required services without catastrophic expenditures.^4^ Roemer and Trannoy [65] note that government health expenditure does not usually benefit the poor due to the unfair distribution of benefits. Some individual and household factors are age, gender, urban–rural differences, marital status, socioeconomic status, household size, employment, education, and health insurance. Policymakers can achieve greater equality in service delivery by addressing the availability, affordability, and acceptability of health services either directly or via policies to boost the individual characteristics that could improve access, such as educational attainment and employment.

Focusing on individual and household factors of access, Anselmi et al. [66] found that health and healthcare services inequality was strongly related to household socioeconomic indicators in Mozambique. Affluent households are more likely than poor households to access healthcare services, because they have more financial resources than the poor to acquire services, even if these are far away or are offered by the more expensive private sector. Uninsured, unemployed, uneducated, and rural populations are also likely to have weak access to healthcare services due to high out‐of‐pocket expenditures and transport costs. Ataguba and Goudge [67] found that those with health insurance are more likely than the uninsured to incur high out‐of‐pocket expenditures as they make more use of private healthcare facilities and have to make co‐payments for facilities not covered by insurance.

Urban–rural differences also affect access to healthcare services. Rural areas are more likely to be underdeveloped, with rudimentary infrastructure and further travel to the nearest facility, increasing travel time and cost, thereby reducing access to healthcare services. Gilson and McIntyre [11] note that rural and urban populations have unequal access to healthcare services, as rural facilities frequently struggle with shortages of health workers, equipment, and drugs. Transport availability remains a problem even when healthcare services are free [30], particularly since rural people have limited access to private vehicles.

Burger and Christian [27] found that men, educated and younger people, are more likely than women, uneducated and older people, to be satisfied with access to healthcare services. Marital status and household size also affect access to healthcare services, with married individuals and small households having better access than unmarried individuals and large households.

We used the studies reviewed as a background for our investigation of inequality in access to healthcare services in Zimbabwe. We looked at two dimensions: availability and affordability. We did not have data to examine the third dimension, acceptability.

**Inequity Versus Inequality in Healthcare**

**Inequity**

Equity refers to treating people with different circumstances differently to ensure similar outcomes for all. According to luck egalitarianism, individuals with compromised circumstances should receive additional benefits to offset these circumstances [65]. Therefore, equitable distribution of resources should result in those worse off being granted, through a redistribution of resources, opportunities equal to those of the wealthy. However, the notion of luck egalitarianism is criticized by Ekmekçi and Arda [68] and Anderson [69] for focusing only on the results of an individual's choices, in this case related to an individual's health status, with little regard for systems and processes that may cause such inequity, such as inequality in the distribution of resources. In contrast, relational equity focuses on changing the social norms that contribute to inequality and changing the structure of public goods to reduce inequality, and not the redistribution of resources [69].

Although equal opportunity is not morally acceptable when compared to Anderson's [69] relational equity, it is important to note that people with the same conditions are supposed to be treated equally. Gulliford et al. [70] also stress that to achieve equity in access to healthcare services, people with the same need should be given equal access to such services. The luck egalitarian theory is, therefore, useful in explaining the resource redistribution to achieve equity in health. The poor tend to have more healthcare needs than the affluent, and the egalitarian view of equity favors the distribution of health resources towards them. Besides socioeconomic status, equity is also considered for people of different age groups, gender, and urban–rural differences. It is difficult to measure inequity directly due to inadequate information on healthcare needs in the household survey. Therefore, inequity is often inferred using measures of inequality.

**Inequality in Health**

Health inequality reflects disparities in health between different groups, and equality entails treating everyone the same. Adam Wagstaff, a well‐known researcher in the field of health inequalities, noted that there are disparate health outcomes between groups in both developed and developing countries [71]. These outcomes may be related to inequalities in the healthcare services provided or to social determinants of health. Poor sanitation, unhealthy living and working environments, poor nutrition, and the prevalence of communicable and noncommunicable diseases threaten the health of the majority in developing countries [72, 73]. Research on health inequality has, therefore, gained momentum in developing countries [71].

Literature on health disparities in developing countries mainly focuses on child health outcomes. These child health outcomes include infant mortality, under‐five mortality, malnutrition, diarrhea, and acute respiratory infections. Existing literature notes that these child health outcomes are prevalent in developing countries, and mainly affect the poor [71, 73, 74]. Wagstaff [71] noted that several factors contribute to health disparities in developing countries. These factors are discussed below.

Wagstaff [71] noted that income disparities are associated with concomitant health disparities, but found this relationship to be insignificant in developing countries, while Wagstaff and Van Doorslaer [75] found a weak relationship between the two variables. Contrary to Wagstaff [71] and Wagstaff and Van Doorslaer [75], Van Doorslaer et al. [74] noted that income disparities are positively and significantly related to health outcomes in developing countries.

Another factor is differences in average income per capita. The relationship between income per capita and health outcomes can be explained in different ways. Literature notes that an increase in income per capita increases health disparities, given that income elasticity increases as income increases [57, 71]. The affluent are more likely to have the same health disparity as the poor if the income elasticity does not change with income. On the other hand, health disparity is less evident amongst the affluent compared to the poor if income elasticity changes are less than the changes in income. Consistent with Wagstaff [71], Lleras‐Muney [73] notes that low socioeconomic status is associated with poor health outcomes. Affluent areas are, therefore, likely to have more health disparities than poor areas. Surprisingly, more health disparity is experienced in areas with strong economic growth, due to technological advances in these areas, which the affluent use to their advantage [71]. In the long run, the average income ceases to influence mortality, regardless of economic development; what matters is the income disparity [72, 76].

Public health expenditure is another factor associated with health disparities in developing countries [57, 71]. Health expenditure enables the provision of subsidized or free healthcare services. It improves the health of both the affluent and the poor, but the health of the poor shows greater improvement [57, 71]. However, both Wagstaff [57, 71] and Van Doorslaer et al. [47, 74] found the effect of increased spending on reducing disparities to be statistically insignificant, possibly because subsidies also benefit the affluent.

Wagstaff [71] and Deaton [72] note that methods such as cross‐country comparative studies, country‐time comparative studies, benefit incidence analysis, and decomposition analysis aid in assessing and devising policies that support the reduction of health inequalities.

## Appendix B1 Robustness checks

See Tables B1, B2, B3.

**Table B1:** Chi‐square test of association.

	Urban	Rural	Total	Pearson *χ* ^2^	*p* value
Affordability					
Individuals with proportion of health and per capita nonconsumption expenditure < 40%	723	4010	4733	54.863	0.000
	(0.057)	(0.314)			
Individuals with proportion of health and per capita nonconsumption expenditure ≥ 40%	870	7188	8058		
	(0.068)	(0.562)			
Availability					
Individual's traveling distance < 5 km	725	8766	9491	608.798	0.000
	(0.052)	(0.63)			
Individual's traveling distance ≥ 5 km	989	3422	4411		
	(0.071)	(0.246)			

**Table B2:** CI for fiscal incidence using equivalence scales.

	OECD‐modified scale	OECD equivalence scale	Square root scale			
	Public clinics	Public hospitals	Public clinics	Public hospitals	Public clinic	Public hospital
Urban	−0.287*, **	−0.075	−0.287***	−0.099	−0.323***	−0.021
	(0.083)	(0.099)	(0.083)	(0.073)	(0.084)	(0.102)
Rural	−0.121***	0.046	−0.121***	−0.186	−0.117***	0.148
	(0.010)	(0.081)	(0.010)	(0.225)	(0.010)	(0.108)
Total	−0.201***	0.296*	−0.201***	0.169*	−0.198***	0.315***
	(0.012)	(0.109)	(0.012)	(0.098)	(0.012)	(0.117)

*Note:* Robust standard errors are in parentheses.

*** *p* < 0.01;

* *p* < 0.1.

**Table B3:** CI for access to health care services using equivalence scales.

	OECD‐modified scale	OECD equivalence scale	Square root scale			
	Affordability	Availability	Affordability	Availability	Affordability	Availability
Urban	0.195***	−0.086	0.195***	−0.086	0.180***	−0.091
	(0.064)	(0.055)	(0.064)	(0.055)	(0.063)	(0.055)
Rural	0.145***	−0.032	0.145***	−0.032	0.127***	−0.037
	(0.022)	(0.023)	(0.024)	(0.023)	(0.024)	(0.023)
Total	0.095***	0.078***	0.095***	0.078***	0.082***	0.073**
	(0.028)	(0.029)	(0.029)	(0.029)	(0.028)	(0.029)

*Note:* Robust standard errors are in parentheses.

*** *p* < 0.01

** *p* < 0.05.
